# Ratings or Sales? The Neural and Psychological Processes of Online Experience Product Purchase: Evidence from a Sample of Chinese University Students

**DOI:** 10.3390/bs12120499

**Published:** 2022-12-07

**Authors:** Keyu Chen, Wuke Zhang, Pengtao Jiang

**Affiliations:** 1 Business School, Ningbo University, Ningbo 315211, China; 2 Nottingham University Business School China, University of Nottingham Ningbo China, Ningbo 315100, China; 3 School of Information Science and Engineering, NingboTech University, Ningbo 315100, China

**Keywords:** product ratings, monthly sales, extrinsic cues, online cues, experience product, event-related potentials (ERPs)

## Abstract

Extrinsic cues are ubiquitous in daily commodity consumption scenarios, not to mention online consumption scenarios. Among the many online cues, monthly sales and product ratings are two of the most representative. Some scholars have researched the impact of these cues on consumer decision making, but only search products have been investigated. Based on previous research, this article expanded the types of products to experience products and further explored consumer purchase behaviours and the underlying purchase processes influenced by these two extrinsic cues with the assistance of a neuroscience tool, event-related potentials (ERPs). The behavioural results indicated that the subjects decided mainly based on ratings, while the effect of sales was continuously inhibited. The ERP results further suggested that consumers recognised low ratings and low sales as more negative stimuli than high ratings and high sales, as larger P2 amplitudes were observed. Following the early processing of these cues, low ratings were considered unacceptable and evoked more significant emotional conflicts than high ratings, which was reflected by larger N400 amplitudes. Moreover, in the late stage, high ratings, which activated evaluation categorisation and produced more significant emotional arousal than low-rating conditions, guided the formation of purchase intention and reflected greater LPP amplitudes. Theoretical and managerial implications were discussed.

## 1. Introduction

Recently, a report showed that global e-commerce sales once again hit a record high [1]. With the further development of e-commerce, online shopping has proliferated and become an essential part of people’s daily lives. However, limited by time and space, consumers cannot directly observe products when shopping online, which inevitably brings about information asymmetry [2] and makes product quality more uncertain [3]. In such cases, consumers are more likely to use cues provided by online stores to reduce uncertainty and assess product quality [4].

According to the cue utilisation theory [5], products deliver a series of cues, which can be divided into extrinsic and intrinsic cues and are commonly used by consumers to evaluate product quality [6]. Intrinsic cues regard the direct attributes of the product (e.g., product specifications), and extrinsic cues are related to indirect attributes (e.g., sales volumes). Previous studies suggest that consumers are more likely to use extrinsic cues online to evaluate product quality when they cannot physically observe the intrinsic cues of products [4]. Thus, this study employs two vital and unique extrinsic cues in online shopping: monthly sales and product ratings (for short, sales and ratings are used below). Online stores frequently use sales, which have shown to play a vital role in guiding consumers to better place an order [7]. Moreover, sales have long been reported to influence purchase intentions through the herding effect [8]. Ratings, which are the simple version of consumer reviews [9], have shown to have a significant influence on consumers’ perception of product quality [10] and ultimately impact purchase intentions to a great extent [11,12,13]. 

A previous study researched the impact of two online cues, sales volumes and product ratings, on consumer purchase decisions, but only headsets were used as the experimental material [9]. Commonly, products can be classified as search or experience [14,15]. Search products represent products whose attributes can be assessed objectively even before purchase using second-hand data. In contrast, experience products represent products whose attributes can only be assessed after purchase and in person [14,15,16]. The headset, which was used by Wang, Meng, Liu, Wang and Ma [9], is commonly distinguished as a search product. Previous studies highlight that consumers have different information processing procedures when purchasing search and experience products [17,18]. In other words, due to consumers’ lack of ability to assess experience product quality and reduce the risk of information asymmetry when shopping online, consumers may rely significantly more on rating information to make purchase decisions (compared with search products). Therefore, this study aimed to address the following questions: (1) do experience products influence consumer online purchase intention based on ratings and sales cues? (2) If so, what specific influence occurs? (3) What are the neural and psychological processes of experience product purchase? 

Building on the basis of the cue utilisation theory, the cue diagnosticity framework (shortly called framework below) proposes that when consumers are faced with multiple extrinsic cues simultaneously, they intuitively classify cues according to the perceived reliability (diagnosticity) of cues [19]. The results of the classification are noted as high-scope cues (perceived to be more reliable) and low-scope cues (perceived to be less reliable) [20]. The framework suggests that the negative (positive) inferences on perceived product quality elicited by high-scope cues pass to low-scope cues, making low-scope cues less (more) diagnostic (original mechanism) [19]. Meanwhile, some studies report that high-scope cues (no matter whether positive or negative) may continuously restrain the influence of low-scope cues on purchase decision making (alternative mechanism) [21,22]. While Wang and her colleagues’ study, using a search product, provides support for the original mechanism of the framework [9], the current study, employing experience products as the key moderator, presents an interaction between sales and ratings that supports the alternative mechanism. 

To address the difficulty in clarifying a vital but nuanced mechanism difference in the framework and simultaneously investigate the neural and psychological processes of experience product purchase, the current study employed event-related potentials (ERPs). Accordingly, the ERP method has the following advantages: First, people lack the ability to either predict their future behaviours or identify their internal mental states through self-reporting accurately [23]. Evidence indicates that traditional self-reporting methods might provide inaccurate data [24]. Pioneering studies also report that explicitly asking participants to self-report such internal mental states and processes leading to choices is considered to alter the outcome of their judgments [23,25]. Secondly, the ERP approach has been proven to have a high temporal accuracy in explaining the neural bases of online shopping decision-making processes [26,27]. Thirdly, as suggested by Ariely and Berns [28], and Boksem and Smidts [29], brain data are less noisy than data obtained with conventional marketing methods, and neuroscience methods can provide marketing researchers with information that cannot be obtained through traditional methods. From this perspective, the ERP method can finely unveil the psychological processes of experience product purchase at the brain level and provide precious evidence for our behavioural argument. 

The current study makes at least the following contributions: First, it contributes to the “online purchase” literature and cue diagnosticity framework by providing additional support to the alternative mechanism of the framework using experience products as experimental materials. Second, it provides additional verification of the ERP results and explanations of the valuable previous study [9], suggests a supplementary explanation of the LPP component, and further infers the existence of a dual process. Third, it proposes clear guiding significance for online shopping practice, especially for online retailers.

The following paper is organised as follows: First, we review the relevant literature and subsequently propose hypotheses. Secondly, we describe our ERP experimental materials and method. Then, behavioural and ERP results are presented. Finally, we discuss the key findings, theoretical contributions, managerial implications, and research limitations.

## 2. Theoretical Background and Hypothesis Development

### 2.1. Online Cues and Cue Diagnosticity Framework 

As mentioned in the Introduction section, the cue utilisation theory proposes that a product brings a series of cues that can be categorised as intrinsic (representing product-related attributes, e.g., parameters of digital devices) and extrinsic (representing later-added (indirect) product information, e.g., consumer feedback) [5,6]. Previous studies consistently suggest that these cues, such as the visual attractiveness of webpages [4], warranties and guarantees [22], and online consumer reviews [21,30], are the basis for online purchase decisions [22,31]. Evidence also shows that consumers are more likely to use extrinsic cues to evaluate product quality when it is hard to physically examine products (which means that intrinsic cues are hard to obtain) [4]. Among various online extrinsic cues, consumer reviews and sales volumes have been extensively investigated [9,10,21,30]. The present research also focuses on these two cues: product ratings (a simplified version of consumer reviews) and monthly sales (a common way to display sales volumes in online shopping platforms). In the following paragraphs, arguments in favour of these two cues are detailed.

Product ratings are the quantitative evaluation of all product reviews and are usually demonstrated on a 5-point Likert scale (with “1” as the lowest and “5” as the highest). Past research suggests that ratings share some essential features with consumer reviews [9,11]. Based on the previous study’s [9] proposal, we believe that at least the following feature is shared: consumer reviews are reported to have an impact on consumer perception to a great extent [9]. Concretely, since they are the feedback directly posted by other consumers, they are considered an efficient way to reduce information asymmetry, and their characteristics are regarded as cues reflecting product quality [10]. Furthermore, ratings have been directly proven to increase perceived quality and purchase intention by mitigating risks in the e-commerce context [11]. Thus, reviews (as well as ratings in the current study settings) can provide abundant product-related information and significantly influence consumer-perceived product quality, which is of great importance for purchase decision making in e-commerce [9,12,13]. 

Monthly sales, which online stores frequently use, are also essential and unique extrinsic cues [7]. They are defined as the number of products ordered in the latest month. Sales volume information has long been examined in the marketing literature (see Ali, et al. [32] for a review). A research study using online shopping settings demonstrated sales’ significant effect on consumer purchase intention, and the effect was attributed to the “herding effect” [8]. The herding effect indicates that people are inclined to obey the guidance and choices of the majority and adopt behaviours consistent with that of others under insufficient information conditions [8,33]. In summary, although sales volume may not bear direct product quality-related information, it can still influence consumer purchase intention by activating herding behaviour [8].

Moreover, consumers are commonly exposed to multiple extrinsic cues simultaneously when shopping online, so the interactive effect among these cues should be considered. The present study tries to unveil this interaction by employing the cue diagnosticity framework. As mentioned in the Introduction section, the framework suggests that consumers tend to process and classify cues intuitively based on their diagnosticity in discerning among alternative products [18]. Past research distinguishes the classification results as high-scope cues, which are highly related cues that evolve without instantaneously changing in valence, and low-scope cues, which are low-relevance cues whose valence changes relatively frequently [20]. In other words, high-scope cues should be perceived as more reliable (continuing to indicate a highly related stable attribution) and, consequently, more diagnostic than low-scope cues. Taking online shopping settings into consideration, when consumers are exposed simultaneously to both sales and ratings, although sales may indicate the majority’s choice and lead to herding behaviours [8], a rational consumer’s priority (without impulsive motivation [34] or time pressure [35], which may reform consumer behaviour but is beyond the scope of this research) should be reducing information asymmetry and assessing product quality. Thus, ratings, which bear product quality-related information [10,12] and are relatively hard for sellers to manipulate [9], rather than sales should be processed as high-scope cues by consumers. 

The framework further states that high-scope cues have direct and indirect effects on the impression formation of product quality [20]. While the direct impact is manifested in the formation of strong positive impressions in consumers, as high-scope cues are more stable and credible, the indirect impact is reflected in either the enhancing or weakening of the diagnosticity of low-scope cues and the altering in their usage in product quality judgment. Specifically, positive (negative) high-scope cues facilitate (inhibit) the expression of the diagnosticity of low-scope cues. This framework is supported by many studies alike [9,19]. However, some studies present an alternative mechanism to the cue diagnosticity framework: high-scope cues (positive or negative) may consistently weaken the influence of low-scope cues on making purchase decisions [21,22]. In other words, consumers may rely more on high-scope cues to make a purchase decision, and the effect of low-scope cues is constantly inhibited. For example, Utz, Kerkhof and Van Den Bos [21] report that as the effect of factors such as assurance seals (low-scope cues) is prominent when presented with consumer reviews (high-scope cues), the impact of various cues is not combined in a simple additive manner. Although our inferences on ratings (high-scope cues) and sales (low-scope cues) in the current settings are consistent with Wang’s study [9], moderators (e.g., contextual variables) that can alter the underlying mechanism (either following the original cue diagnosticity framework or the alternative one) may still exist. In the next section, we propose a potential moderator: product type.

### 2.2. Product Type Theory

Many classification schemes for products have been proposed in previous research [36,37,38]; this study adopts the search and experience classification [14,15]. This classification method has been argued to be a valuable way for evaluating the potential of products through the Internet as a marketing channel [16,30,38,39]. We define search and experience products by following the classic literature [14,15,16] with updates related to the contemporary context. A search product is a product that consumers can inspect for quality either in person or using second-hand data that specify detailed specifications, and the inspection must occur before purchasing the brand, while an experience product is a product whose inspection becomes too expensive and that consumers can only inspect for quality in person after purchasing (expensive means that more effort, whether monetary, cognitive, or time, is needed). 

Based on the definitions and discussions in the previous literature, experience and search products show at least three obvious differences: Firstly, in terms of “time”, while the quality of the search product can be assessed before purchase, the quality of the experience product can only be inspected after purchase [14,15]. Secondly, in terms of “method”, the quality of the search product can be evaluated using second-hand data, but the quality of the search product can only be evaluated in person [14,15]. Thirdly, in terms of “effort”, the effort for consumers to determine product quality is higher for experience products than for search products [14,15]. In general, when consumers buy an experience product, only after the purchase, through personal experience, and after having made a much higher effort than for a search product, can the consumers determine the quality of the product, which has been shown to have a high correlation with purchase intention [13] and satisfaction [40]. Furthermore, previous studies have proved that consumers show significantly different information processing procedures and buying habits when they purchase search and experience products online under actual circumstances [17,18,41,42]. For example, in the study by Senecal and Nantel [42], participants were found more likely to select the product recommended by others when choosing an experience good (wine) than when choosing a search good (calculator). In another study, Huang, Lurie and Mitra [17] discovered that consumer feedback increased purchase intention, which was more significant for experience than search goods.

Generally speaking, the same set of information may cause completely different buying decisions for search and experience products. Thus, we propose that in the online experience product purchase context, ratings (high-scope cues) bearing product quality-related information catch consumers’ particular attention and continuously inhibit the presentation of the diagnosticity of sales (low-scope cues).

### 2.3. Behavioural Hypotheses

Both sales and ratings can seemingly manifest the perceived quality of products and influence consumer judgment and purchase intention towards them. However, this influence seems to vary between search and experience products [17,42]. When sales appear alone, studies have proven that people are more willing to buy products because of the herding effect [8,17]. When ratings appear as a single extrinsic cue, they can be used as a signal to reflect product quality, reduce perceived risks, and significantly affect consumers’ perception of product quality [11,42]. This feature of ratings coincides with the fact that consumers need more product quality-related information to help them to form subjective product quality perceptions before purchasing experience products [43,44]. When sales and ratings appear simultaneously, they inevitably trigger an interactive effect [9]. Based on the previous discussion, we predicted that for experience products, ratings have more significant impacts on purchase intention than sales. Moreover, compared with sales, ratings are harder to be manipulated by e-retailers; thus; they are likely to be more stable and credible. Therefore, consumers intuitively believe that ratings are more diagnostic, regard ratings as high-scope cues, and classify sales as low-scope cues. In brief, ratings directly and significantly impact the purchase intention of experience products and indirectly affect purchase intention by inhibiting the usage of sales in the decision-making process.

Thus, we developed the following behavioural hypotheses:

**H1a.**  *In experience product purchasing, high ratings and high sales induce the highest purchase intention, and low ratings and low sales induce the lowest purchase intention*.

**H1b.**  *In experience product purchasing, ratings have a greater impact on purchase intention than sales*.

### 2.4. ERP Method

In recent years, various tools and approaches appeared in marketing research, for example, eye tracking, fMRI, and ERPs. In the present study, we aim to clarify a vital but nuanced mechanism (of the cue diagnosticity framework); another goal is that of unveiling the neural and psychological processes of experience product purchase. Amongst these tools, event-related potentials (ERPs) can offer insights into the cognitive processes at the brain level with high temporal resolution, which makes this a more suitable measure to track rapid temporal modulations in neural activities [45]. Thus, we employed ERPs in this research. Previous studies employing the ERP method have shown its value in exploring the cognitive process in multiple dimensions, particularly underlying emotions, motivations, and conflicts [26,27,46]. More specifically, lots of researchers have used the ERP method to detect brain responses to supplementarily explain specific consumer behaviour [47] and better understand the efforts of marketing-related stimuli such as product photos, reviews, and brands [27]. Thus, it was possible for us to conduct a study with ERPs to investigate how online cues influence the cognitive and decision-making processes of experience product purchase.

Considering that consumers are expected to experience a series of cognitive processes while shopping online, the current study mainly focused on three frequently investigated ERP components that are closely related to attention distribution (P2), emotional conflicts (N400), and evaluation categorisation and emotional arousal (late positive potential; LPP).

### 2.5. ERP Components and ERP Hypotheses

#### 2.5.1. P2 Hypothesis

P2 is a positive-going component that usually peaks around 200 ms after stimulus onset. It generally appears in the frontal region of the scalp and presumably reflects the intuitive assessment of stimuli [48]. Previous studies consistently indicate that negative stimuli allocate more attention resources than positive stimuli and elicit a larger P2 amplitude [49,50]. For instance, in the study by Bublatzky and Schupp [49], when participants detected the hazard while evaluating warning signal words, a greater P2 amplitude was elicited.

Since P2 reflects the automatic allocation of attention and is very sensitive to negative stimuli, in the current study, we supposed that low sales and low ratings were identified as negative stimuli and strongly affected the P2 amplitudes. Thus, the following hypothesis was proposed:

**H2.**  *In experience product purchasing, low monthly sales and low product ratings trigger larger P2 amplitudes than high monthly sales and high product ratings, respectively*.

#### 2.5.2. N400 Hypothesis

N400 is a negative-going component of ERPs observed approximately 400 ms after stimulus onset [51]. Past studies have reported that conflicts related to semantic meaning often elicit large N400 amplitudes [51,52]. Recently, some studies have suggested that N400 is the reflection of other conflicts. For example, in neuroscience, researchers have shown that N400 can reflect the deviation and differences between exposed and acceptable information [53,54]. From this perspective, more recent studies have reported that N400 is sensitive to emotional conflicts [55,56]. Specifically, the greater the deviation between acceptable and exposed information is, the larger the emotional conflicts and N400 amplitudes are [53,56,57].

Based on the discussion above, we inferred that, in this study, low ratings represented a greater likelihood of poor experience product quality and could not be accepted by consumers, leading to obvious emotional conflicts and N400 amplitudes. On the other hand, although sales are correlated with herding behaviours [8] and might cause deviations between low sales and acceptable information for consumers, which may lead to potential emotional conflict, after considering the interaction between sales and ratings and the characteristics of experience products discussed above, we considered that when purchasing experience products online, ratings are high-scope cues that inhibit the expression of the diagnosticity of sales. In other words, when the ratings are low, consumers may not accept the product regardless of the high or low sales; when the ratings are high, consumers may rely more on this information and directly show acceptance.

Thus, we developed the following hypothesis:

**H3.**  *In experience product purchasing, compared with high product ratings, low product ratings (regardless of monthly sales) elicit greater N400 amplitudes*.

#### 2.5.3. LPP Hypothesis

The late positive potential (LPP) is an ERP component of the central–parietal regions and typically peaks approximately 600 ms after the presentation of a stimulus [58]. Since highly emotional arousal stimuli such as emotional text [59], affective pictures [46], and facial expressions [60] are reported to elicit greater LPP amplitudes, the LPP is considered to be correlated with emotional arousal, as reported by psychophysiology and neuroscience studies in the past decade [46,59,60]. More specifically, it is considered as a reflection of motivational, emotional arousal and a signal of underlying motivations of consumer behaviour [61,62]. For example, the results of Zhang, Jin, Wang, Ma and Yu [27] study showed that along with the fulfilment of social goals of self-presentation after purchasing luxury goods, larger LPP amplitudes were detected. In the current study, the ERP experiment simulated the scenario of consumers browsing online to purchase products. The ultimate goal of the participants in each trial was to evaluate the product purchase intention and make a satisfying purchase decision. Studies show that completing a satisfying consumer purchase could excite personal goal achievement emotions [63,64].

Moreover, an alternative explanation exists for the LPP component. Recent decision studies have also found LPP amplitudes to be positively related to cognitive evaluation categorisation [26,56,65,66], which is based on intuitive psychological categorisation models using similarity [67] as a central classification factor [9]. The higher the category similarity is, the larger the LPP amplitudes are. Using previously formed, acceptable (desirable) extrinsic cues, consumers may categorise present cues based on their similarity with acceptable cues (i.e., the closer the present cues are to the acceptable cues, the higher the similarity is). Since high ratings (compared with sales) indicate acceptable product quality to a greater extent and given their possible inhibition effect on sales, high-rating conditions may be perceived as more similar to the acceptable cues by the consumer, reflecting larger LPP amplitudes.

However, these seemingly distinct explanations are, in fact, not conflicting. Both of these inferences have been drawn by scholars based on the same physiological basis, i.e., attentional resource distribution (the more attentional resources the participants capture, the larger the LPP amplitude is) [68]. Unfortunately, the current development of measurement techniques forbids us from clearly distinguishing whether in the online experience product purchase, consumers experience a process of emotional arousal or evaluative categorization (for a further discussion on the limitations of the ERP technique, see the Limitations and Future Research section). However, that does not prevent us from proposing a hypothesis about the LPP amplitudes. Thus, based on the discussions above, we hypothesized the following:

**H4.**  *In experience product purchasing, compared with low product ratings, high product ratings (regardless of monthly sales) elicit greater LPP amplitudes*.

## 3. Materials and Methods

### 3.1. Subjects

To estimate how many subjects were needed, we performed a power analysis using G*Power 3.1 [69]. Vazire [70] advised researchers to ensure that the power value does not fall below 80%. Thus, we entered the parameters as follows: an effect size of 0.25 (medium size), an alpha level of 0.05, and a power value of 0.8, and we left the within-subject measurement correlation (0.5) and the non-sphericity correlation value (1) as default. The result of the power analysis estimated a sample size of 24. Therefore, 30 native Chinese students (12 males and 18 females) were recruited at Ningbo University. Their ages ranged from 19 to 24 years old (M=20.83, SD=0.95), and all of them had online shopping experience. All participants were students with normal or corrected-to-normal visual acuity. They self-reported as right-handed and had no history of neurological disorders or mental diseases. Subjects were paid RMB 40 (around USD 6) for their participation. One of the participants was excluded due to excessive artefacts in the electroencephalogram (EEG) recordings. Ultimately, the number used for data analysis was 29 (11 males). The study was approved by the Internal Review Board of Academy of Neuroeconomics and Neuromanagement at Ningbo University (approval code: BGP20210619).

### 3.2. Materials

The target stimulus materials were 5 experience products. To determine the experience products used in the formal experiment, 52 questionnaires were sent to Chinese college students, and 43 valid replies were received. Consistently with a former study [42], the definitions of experience and search product were shown in the questionnaire. Subjects were asked to evaluate and consider the experience attributes of each candidate product and scored on a 5-point Likert scale ranging from 1 (very inconsistent with the definition) to 5 (closely consistent with the definition). The results of the five experience products with the highest scores were as follows: facial cleanser (M=4.52), T-shirt (M=4.40), chocolate (M=4.38), sunglasses (M=4.30), and peaked caps (M=4.29). We also conducted a *t*-test between the products selected and the 5 products with the lowest scores, and the result was significant (t=6.125, p < 0.001). To eliminate the feeling of repetition and fatigue, we prepared 8 different photos for each kind of product (40 pictures in total), which were randomly and evenly divided into four blocks (40 trials each, corresponding to four conditions) in the formal experiment. All the pictures were selected from tmall.com, and each picture was shown on a white background with a size of 270 × 360 pixels. Irrelevant information was eliminated using Photoshop 2019 (Adobe Systems Incorporated, San Jose, CA, USA).

The current study adopted a 2 (sales: high vs. low) × 2 (ratings: high vs. low) within-subject repeated measures design. To determine the standards for high versus low ratings and sales, we referred to a previous study [9] and made appropriate modifications based on the data on tmall.com. According to the data, the ratings had not changed much, so we still adopted low ratings ranging from 2.00 to 2.25 and high ratings ranging from 4.75 to 5.00, with 1.00 referring to the lowest score and 5.00 corresponding to the highest score [9]. High versus low sales were determined using real sales information on tmall.com and using the same method used in the previous study [9]. As a result, high sales were manipulated to be around 6500 (±7.5%), and low sales were manipulated to be around 100 (±7.5%).

### 3.3. Procedures

**Figure 1:**
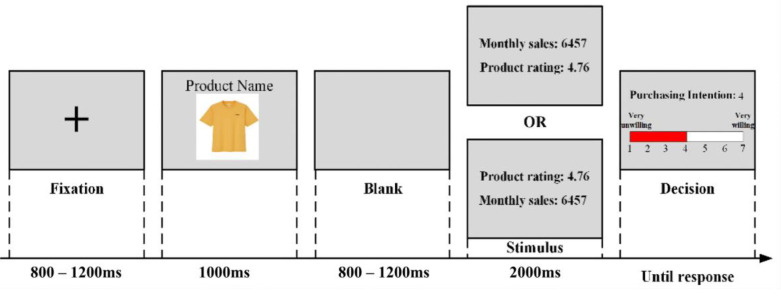
Experiential procedure.

In the experiment, the subjects were asked to enter a soundproof room and sit on a comfortable chair. The chair was 100 cm away from a computer-controlled monitor (1280 × 1024 pixels) with a refresh rate of 60 Hz. Before the formal experiment started, each participant received a paper introducing the task, procedure, and pre-experiment announcements. They were explicitly told before imagining that they were browsing an online shopping platform and planning to purchase products. Stimulus presentation and data collection were controlled using E-Prime 3.0 (PST; Psychology Software Tools, Inc., Pittsburgh, PA, USA). As shown in Figure 1, each trial began with a black cross against a grey background for 800–1200 ms. Then, we shortened the display time of the product picture and its name to 1000 ms, hoping that the participants could pay more attention to the subsequent sales and rating information. A blank of 800–1200 ms was added before the cue stimuli appeared to facilitate future data analyses, since a whole epoch usually needed 200 ms before stimuli as baseline correction. After that, stimuli containing both ratings and sales information were presented for 2000 ms. The relative position of ratings and sales cues was counterbalanced to eliminate the potential influence of reading order. Then, the participants could use the keypad provided to report their purchase intention through an on-screen Likert scale ranging from 1 (very unwilling) to 7 (very willing). Participants were asked to minimise blinks, eyes, and head movements during the experiment. The formal experiment started after 8 practice trials.

The following details about the experiment need to be explicitly explained: (1) the price of each product was controlled to be similar (around USD 10); (2) subjects were told before the experiment that the purchasing decision for each product had to be made independently; (3) during the interval of each block, subjects could take a short break, and the whole formal experiment lasted around 20 min.

### 3.4. Behavioural Data Recording and Analysis

For the behavioural data, we calculated the mean value of the purchase intention for each condition, followed by a 2 (sales: high vs. low) × 2 (ratings: high vs. low) repeated measures ANOVA. We conducted a simple effect analysis to assess whether there was an interaction effect between the two factors. Additionally, we used partial eta-squared ($\eta^{2}{}_{p}$) values to demonstrate the effect size in ANOVA models, where 0.05 represented a small effect, 0.1 represented a medium effect, and 0.2 represented a large effect [71].

### 3.5. Electroencephalograph (EEG) Recording and Analysis

A 64 Ag/AgCl electrode cap and Neuroscan Synamp2 Amplifier (Curry8, Neurosoft Labs, Inc., Centennial, CO, USA) were used to record EEG data at a sample rate of 500 Hz. The left mastoid was used for reference, and a cephalic location between PFz and Fz was used as the ground. Data were transferred offline to the average of the left and right mastoid references. The electrooculogram (EOG) was recorded using electrodes placed at 10 mm from the lateral canthi of both eyes (horizontal EOG) and above and below the left eye (vertical EOG). EOG artefacts were corrected offline for all subjects. The electrode impedances were controlled below 5 kΩ during the whole experiment. EEG data preprocessing was performed using the EEGLAB toolbox [72] and MATLAB (R2013a; The MathWorks, Inc., Natick, MA, USA). First, EEG data were re-referenced to the average of the left and right mastoids, bandpass-filtered to a range of 0.1–30 Hz, and epoched from −1 s to +2 s surrounding the simulation screen onset; we took the baseline activity from −200 ms to 0 ms preceding the target. An independent component analysis was computed using the EEGLAB toolbox. Then, ICs representing eye blinks or other artefacts were removed from the EEG data. Finally, the EEG recordings from each recording site for each participant were averaged within four conditions (high monthly sales and high product ratings (HS&HR); high monthly sales and low product ratings (HS&LR); low monthly sales and high product ratings (LS&HR); low monthly sales and low product ratings (LS&LR)), respectively.

According to the guideline proposed by Picton, et al. [73] and the visual observation of grand averaged waveforms, three ERP components were analysed, P2, N400, and the LPP. To analyse the mean amplitudes of P2, we chose the time window of 200–220 ms after the onset and included 6 electrodes (FC3, FCz, FC4, C3, Cz, and C4) in the frontal–central area into the statistical analyses [48]. A 2 (sales: high vs. low) × 2 (ratings: high vs. low) × 6 (electrodes) ANOVA was employed in the P2 analysis. Similarly, for N400, a time window of approximately 425–475 ms was selected, with 9 electrodes (C3, Cz, C4, CP3, CPz, CP4, P3, Pz, and P4) in the whole brain area [74]. A 2 (sales: high vs. low) × 2 (ratings: high vs. low) × 9 (electrodes) ANOVA was conducted for the N400 amplitudes. The LPP component was analysed with a time window of approximately 500–560 ms and 6 electrodes (CP1, CPz, CP2, P1, Pz, and P2) in the whole brain area [75]. A 2 (sales: high vs. low) × 2 (ratings: high vs. low) × 6 (electrodes) ANOVA was conducted for the LPP amplitudes. Where there was a significant interaction effect among these factors, a simple effect analysis was conducted. In addition, we also used paired-samples *t*-tests to further verify the main effects and used partial eta-squared ($\eta^{2}{}_{p}$) values to demonstrate the effect sizes in the ANOVA models.

## 4. Results

### 4.1. Behavioural Results

The descriptive statistical results of behavioural data are shown in Table 1.

**Figure 2:**
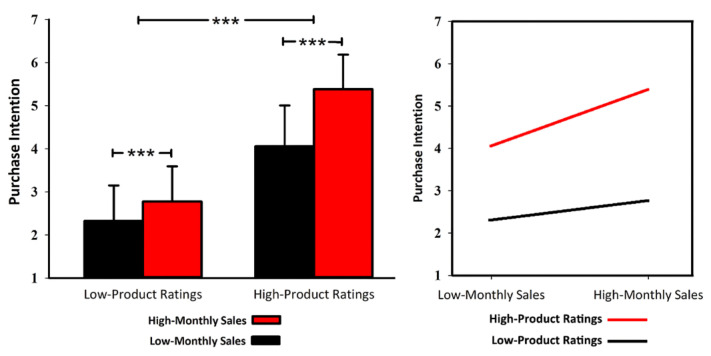
ANOVA and simple effect test results of behavioural data. Note: *** *p* < 0.001.

As shown in Figure 2, the results of the 2 × 2 repeated measures ANOVA indicated significant main effects of both sales ($F\left(1,28\right)=60.050,\;p\;<\;0.001{,\;\eta }^{2}{}_{p}=0.682$) and ratings ($F\left(1,28\right)=108.251,\;p\;<\;0.001{,\;\eta }^{2}{}_{p}=0.794$). The interactive effect between sales and ratings was also significant ($F\left(1,28\right)=23.352,\;p\;<\;0.001{,\;\eta }^{2}{}_{p}=0.455$). As revealed by the results of the simple effect test, under both high- and low-rating conditions, higher sales led to higher purchase intention (high ratings, $M_{\mathrm{HS}}=5.383$ vs. $M_{\mathrm{LS}}=4.057,\;p\;<\;0.001$; low ratings, $M_{\mathrm{HS}\;}=2.773$ vs. $M_{\mathrm{LS}}=2.323,\;p\;<\;0.001$). Thus, H1a was supported. Moreover, from the perspective of the absolute value of mean purchase intention, the final average purchase intention with high ratings was not below 4 (4.057 and 5.383), and low ratings did not exceed 3 (2.323 and 2.773, on the 7-point Likert scale). A pairwise comparison was conducted to support the observation. The results showed that the purchase intention with HS&HR (M=5.383) was significantly higher than that with LS&HR (p < 0.001), HS&LR (p < 0.001), and LS&LR (p < 0.001) and that the purchase intention with LS&HR (M=4.057) was also significantly higher than those under the conditions of HS&LR (p < 0.001) and LS&LR (p < 0.001). Meanwhile, the effect size of ratings ($\eta^{2}{}_{p}=0.794$) was greater than that of sales ($\eta^{2}{}_{p}=0.682$). Thus, ratings showed a stronger impact on the purchase intention, so H1b was also supported.

### 4.2. ERP Results

**Figure 3:**
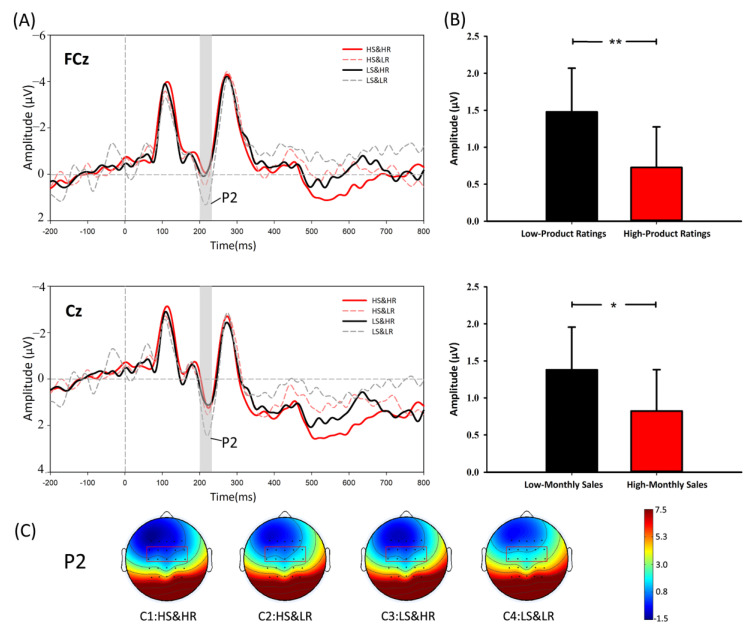
(**A**) Waveform diagrams of P2 component. (**B**) ANOVA results of P2. (**C**) Scalp topographic maps of P2 corresponding to four conditions. Note: * *p* < 0.05, ** *p* < 0.01.

**P2 analysis.** As shown in Figure 3, the results of the 2 × 2 × 6 repeated measures ANOVA for P2 (positive polarity: a larger voltage value meant larger amplitude) indicated that the main effects of both ratings ($F\left(1,28\right)=8.296,\;p=0.08{,\;\eta }^{2}{}_{p}=0.229$) and sales ($F\left(1,28\right)=5.894,\;p=0.022{,\;\eta }^{2}{}_{p}=0.174$) were significant. We also performed paired-samples *t*-tests (HS vs. LS: t=-2.428, p=0.022, LS > HS; HR vs. LR: t=-2.880, p=0.008, LR > HR). The results of the *t*-tests additionally supported the significant main effect of sales and ratings, while the interactive effect between sales and ratings was not significant (F(1, 28)=0.278, p > 0.1). The results of the ANOVA were consistent with our previous assumption. Thus, H2 was supported.

**Figure 4:**
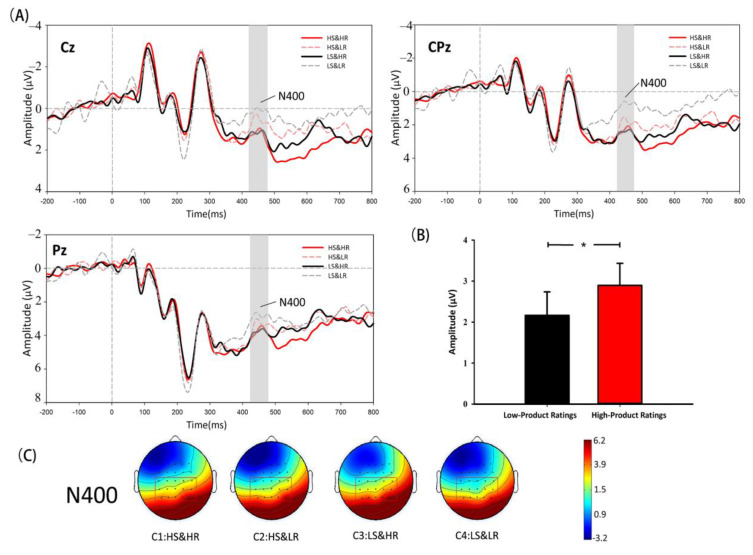
(**A**) Waveform diagrams of N400 component. (**B**) ANOVA results of N400. (**C**) Scalp topographic maps of N400 corresponding to four conditions. Note: * *p* < 0.05.

**N400 analysis.** As shown in Figure 4, the results of the 2 × 2 × 9 repeated measures ANOVA for the N400 amplitudes (negative polarity: a smaller voltage value meant larger amplitude) indicated a significant main effect of ratings ($F\left(1,28\right)=4.733,\;p=0.038{,\;\eta }^{2}{}_{p}=0.145$). The paired *t*-test also supported the result (HR vs. LR: t=2.176, p=0.038, HR > LR). However, for both the main effect of sales (F(1, 28)=0.308, p > 0.1) and the interactive effect between the two factors (F(1, 28)=1.187, p > 0.1), the results were not significant. These results directly supported H3.

**Figure 5:**
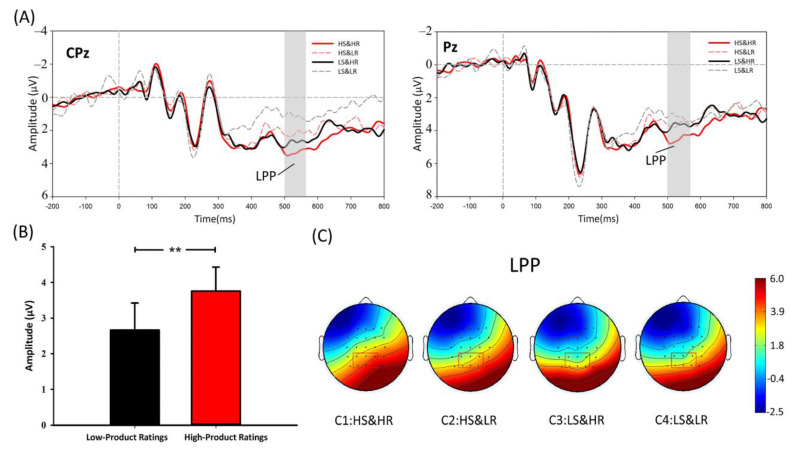
(**A**) Waveform diagrams of the LPP component. (**B**) ANOVA results of LPP. (**C**) Scalp topographic maps of LPP corresponding to four conditions. Note: ** *p* < 0.01.

**LPP analysis.** As shown in Figure 5, the results of the 2 × 2 × 6 repeated measures ANOVA for the LPP (positive polarity: a larger voltage value meant larger amplitude) indicated that ratings had a significant main effect ($F\left(1,28\right)=7.654,\;p=0.010{,\;\eta }^{2}{}_{p}=0.215$). The additional paired *t*-test also demonstrated the main effect of ratings (HR vs. LR:t=2.767, p=0.010, HR > LR). However, there was no significance neither in the main effect of sales (F(1, 28)=3.428, p=0.075) nor in the interactive effect between these two factors (F(1, 28) < 0.001, p > 0.1). Thus, H4 was supported. 

## 5. Discussion

In this study, we investigated consumer behaviours and the underlying brain activities correlated with the influence of sales and ratings on consumer decision making when purchasing experience products online using ERPs.

### 5.1. Discussion on Behavioural Hypotheses

The behavioural results indicated the significant main effects of ratings and sales, and their significant interactive effect. H1a was directly supported. The sample effect test further demonstrated that ratings significantly influenced purchase intention, whether the sales were high or low. Furthermore, from the perspective of the absolute value of purchase intention, although sales significantly affected the subjects’ purchase intention under low-rating conditions, the final average purchase intention score did not exceed 3 (on a 7-point Likert scale). The same effect also appeared under the conditions of high ratings; no matter how high or low the sales were, the average purchase intention score was above 4. This showed that the influence of sales on purchase intention was limited, and it could not reverse purchase decisions towards a particular product. In other words, rating information first determined the tendency of purchase intention (such as “>4” or “<4”). Then, the sales volume affected purchase intention to a relatively small extent in this overall range. Thus, H1b was supported. Based on these results, we argue that consumers are likely to rely more on ratings (high-scope cues) and that the effect of sales (low-scope cues) is continuously inhibited. We also obtained evidence from the ERP results that further supports our argument. 

### 5.2. Discussion on ERP Hypotheses

At the brain level, remarkable P2, N400, and LPP amplitudes were evoked during the purchase decision process. 

#### 5.2.1. P2 Hypothesis

P2 is an early positive component that peaks around 200 ms after stimulus onset and has been suggested to reflect the quick and intuitive processing of stimuli [48]. As reviewed above, P2 is considered to be highly sensitive to negative stimuli [49]. We discovered the main effects of ratings and sales, which provided direct support for H2. The results showed that both low ratings and low sales could elicit greater P2 amplitudes, which suggested that both of these two factors were recognised as negative stimuli by the subjects’ intuitive processing in the experiment. These results indicated that when consumers faced the two online cues, there was an intuitive detection of negative stimuli (negative information).

#### 5.2.2. N400 Hypothesis

Following the P2 component, N400 is considered as the reflection of emotional conflict [55,56]. The stronger the emotional conflict, the larger the amplitude of N400. In the analysis of N400, we found the main effect of ratings. However, neither the main effect of sales nor the interaction between these two factors was revealed (H3 was supported). The existing N400 results suggested that ratings were recognised as high-scope cues and significantly affected consumer decision making by continuously attenuating the effect of sales. As mentioned earlier, in the cue diagnosticity framework, a high-scope cue can either “enable” or “disable” a low-scope cue by altering its diagnosticity. Thus, the emotional conflict caused by violating the guidance of ratings was more intense, and only the main effect of ratings was significant. More specifically, low-rating conditions were considered unacceptable and stimulated strong emotional conflict. It is worth mentioning that, in real online shopping scenarios, between HS&LR and LS&HR conditions, the former is rare, but the latter is sensible, especially when a new product has been recently launched. We speculated that it was also one of the reasons why LS&HR represented a more acceptable condition for the subjects in the experiment.

#### 5.2.3. LPP Hypothesis

The LPP component is recognised as an indication of the distribution of attentional resources [68]. Previous research has further inferred its implications and has suggested its correlation with emotional arousal [46,59,60] and cognitive evaluative categorisation [26,56,65,66]. Consistently with H3, only the ratings’ main effect was found in the LPP amplitudes. However, just as the deduction in the LPP Hypothesis section, the ERPs’ weakness in clarifying “one-to-one” causal relationships is long-lasting [76]. Thus, establishing whether one of these two explanations or their combination truly represents consumer psychological processes of experience product purchase is beyond the capacity of the current research. Indeed, clarifying these two alternative explanations is not the primary goal of the current research and does not influence our most significant findings (showing support for the alternative mechanism of the cue diagnosticity framework in experience product purchase). 

However, we still try to draw some inferences from the results. We propose that a dual process, including an emotional process represented by arousal and a cognitive process represented by evaluative categorisation, exists in the LPP stage. From one perspective, previous studies have described the LPP as a sustained P-300-like component, which may involve a neural process similar to that of P300 [77,78]. Since P300 has been found to be sensitive to both the similarity between expected and displayed information (e.g., [67]) and emotionally significant information (e.g., self-affirming [79] or social rewards [80]), the LPP may also bear these features. This argument, combined with the fact that these two explanations share the same physiological base (attention distribution) [68], further supports that these two explanations are complementary rather than mutually exclusive. From another perspective, previous marketing studies have provided evidence of the co-existence of cognitive and emotional factors in online purchase behaviours (e.g., [81,82,83,84]). Specifically, in Verhagen and Bloemers [83] study, the so-called “think-feel-do hierarchy” of purchase intention was empirically tested in the experience product context. 

Thus, when in a real purchase scenario, emotionally, completing a satisfying purchase after noticing high-rating information excites personal goal achievement emotions in consumers [63], which elicits larger LPP amplitudes. Moreover, cognitively, consumers intuitively categorise products with different ratings into different groups (i.e., “acceptable group with high rating products” and “unacceptable group with low rating products”) based on similarity. A high similarity between high ratings and acceptable cues elicits larger LPP amplitudes. The LPP results were in line with those of N400, which also reflected the special attention that consumers paid to ratings. The effect of high-scope cues (ratings) significantly affected the consumers’ perception of satisfactory consumption (i.e., the generation of personal goal achievement emotions and the perception of high similarity). It also continuously inhibited (or “disabled”) the effect of low-scope cues (sales). Ultimately, only ratings strongly influenced the LPP amplitude. Furthermore, the results of both N400 and LPP additionally supported and explained why the behavioural data showed that ratings had a stronger impact on purchase decisions than sales (H1b).

### 5.3. General Discussion

We summarise our findings and inferences briefly as follows: In a typical experience product purchase scenario, when consumers are faced with sales and rating cues at the same time, first, they quickly and intuitively recognise low ratings and low sales as negative stimuli. Later, affected by the characteristics of experience products, the exposed cues and acceptable cues in the optimal purchase scenario preset by consumers are inconsistent, which causes strong emotional conflicts. In this stage, we speculate that consumers process ratings and sales as high-scope cues and low-scope cues, respectively. Meanwhile, due to the effect of the cue diagnosticity framework, ratings (high-scope cues) suppress the impact of sales (low-scope cues) on the decision-making process. Thus, the N400 component only shows the main effect of ratings. Finally, the effect of high-scope cues continuously exists, and different conditions of ratings (high or low) trigger different extents of personal goal achievement emotions and indicate different categorisations of products based on similarity, elicit different levels of LPP amplitudes, and guide consumers to make final purchase decisions.

### 5.4. Theoretical Contributions and Managerial Implications

This research was carried out under the inspiration of Wang, Meng, Liu, Wang and Ma [9] study and aimed to further explore its topic. Firstly, the current research contributes to the “online purchase” literature and the cue diagnosticity framework by providing additional support to the alternative mechanism of the framework using experience products as experimental materials. Wang and her colleagues’ study [9], using only headphones (a representative search product) as the experimental material, found the interaction between ratings and sales in an additive manner, which fully supported the original mechanism of the cue diagnosticity framework. The current study, using experience products as the moderator (we did not directly test the moderator effect by comparing search to experience products in a single experiment), found a different mechanism whereby high-scope cues (regardless of their positive or negative nature) continuously inhibited the expression of the diagnosticity of low-scope cues, which gave support to the alternative mechanism of the framework. 

Additionally, past research has found other contexts in which the utilisation of cues follows the alternative explanation of the cue diagnosticity framework, for example, consumer reviews vs. store reputation or assurance seals in an online shopping context [21] and Web privacy assurance function vs. transaction-integrity assurance functions in an online vendor context [22]. We contributed to their marvellous findings by adding a new context that supports the alternative mechanism of the framework (i.e., ratings vs. sales in online experience product purchase). 

This theoretical contribution has clear guiding significance for online shopping practice. Specifically, it reminds online retailers that they cannot narrowly focus on managing sales and rating information without considering contextual factors (e.g., the type of products sold in the store). Our study shows that ratings have a greater influence on the purchase intention of an experience product and that the effect of sales is attenuated. If ratings perform worse, sales are unlikely to play a role. From this perspective, online retailers, especially those selling experience products, should build as well as maintain a good-quality signboard in the first place and provide consumers with high-quality products and services. Otherwise, blindly emphasising sales or controlling sales regardless of ratings is meaningless in enhancing consumer purchase intention. 

Secondly, unveiling the brain activities related to online experience product purchase was another important objective in the current study. Considering the small sample size (only 19 subjects were recruited) in Wang’s study [9], the present paper argues that it is necessary to re-examine the neural and psychological mechanisms found in their research study. Therefore, with a comparatively larger sample size and by keeping the experimental settings highly similar, this paper conducted research and discovered the underlying psychological mechanisms of attention distribution (P2), emotional conflicts (N400), and emotional arousal and evaluation categorisation (LPP). These findings were highly similar to those of Wang’s study [9], which further verified our results’ robustness. In addition, we also provide a supplementary explanation of the LPP component and infer the existence of a dual process with the aim to help managers and scholars to appreciate this grey area and better understand the black box of online product purchase.

### 5.5. Limitations and Future Research

The present study has several limitations, which may bring guidance for future research. First, regarding “Other Moderators”, the impact of sales and ratings on product purchase intention in scenarios such as high-value products (often hedonic products) is still unclear and remains to be further discovered. Second, regarding “Results Generalization”, because the ERP experiment has strict requirements for the experimental environment, subjects, and experimental equipment, the online purchase scenario simulated in the experiment is a reduced version of reality and is somewhat different from the real online shopping scenario. Thus, the generalization of the research findings to practice should be made with great care and scrutiny. Third, regarding “Subjects”, although our study recruited a comparatively larger sample than Wang’s study, the sample size was still small. So, we call for re-examination with a larger sample size. In addition, all the participants in the experiment were Chinese students. So, there remain open questions regarding whether the conclusions suit other cultural backgrounds, since people from different countries and nationalities may act divergently when shopping online. Thus, it could be significant for future research to experiment with these online cues from a multicultural perspective. Fourth, despite the increasing popularity of neuroscientific tools in marketing research, scholars should always be aware of their limitations. As shown in our study, one major drawback of ERPs is the interpretation of “one-to-one” causal relationships. Although the current techniques cannot directly clarify the two alternative explanations of LPP components, we hope that the current research successfully shows consumer neuroscience as a complementary link in theoretical systems assessing consumer behaviour from a holistic perspective, including behavioural, physiological, and neural aspects [76].

## 6. Conclusions

This study investigated how online cues influence the decision-making process of experience product purchase using ERPs. As evidence shows that consumers process information differently between search and experience products, we directly focused on this argument as our entry point. As a result, we found differences with respect to previous studies. In experience product purchase, the interaction between high-scope and low-scope cues is attenuating rather than synergistic. Ratings, which are high-scope cues, are more relied upon by consumers to make decisions, and the influence of low-scope cues is continuously inhibited. Furthermore, the results of the ERP analyses showed the brain activities during the cognition process of purchasing. Low sales or ratings were recognised as negative stimuli (P2). Moreover, if the acceptable rating information was violated, significant emotional conflicts (N400) appeared. Last but not least, personal goal achievement emotions were aroused, and evaluation categorisation took place (LPP) if the cues represented high perceived quality, and this guided consumers’ final decision.

## Figures and Tables

**Table 1:** Descriptive statistics and ANOVA results of four conditions.

Product Rating Condition	Monthly Sales Condition	Purchase Intention	S.D. of Purchase Intention	Mean Difference	*p*-Value
High	High	5.383	0.803	1.326	<0.001
	Low	4.057	0.949		
Low	High	2.773	0.819	0.450	<0.001
	Low	2.323	0.826		

## Data Availability

The data presented in this study are available upon request from the corresponding author. The raw data are not publicly available, as they contain individually identifiable information.
